# From the Cochrane Library: Visual Inspection and Dermoscopy, Alone or in Combination, for Diagnosing Keratinocyte Skin Cancers in Adults

**DOI:** 10.2196/41657

**Published:** 2024-03-07

**Authors:** Colleen M Klein, Torunn E Sivesind, Robert P Dellavalle

**Affiliations:** 1 Austin Skin Physicians Austin, TX United States; 2 Department of Dermatology University of Colorado Anschutz Medical Campus Aurora, CO United States

**Keywords:** nonmelanoma skin cancer, dermoscopy, dermatoscopy, teledermatology, dermascopic, dermatoscope, oncology, skin, cancer, basal cell carcinoma, dermatology, cutaneous squamous cell carcinoma, diagnostic odds ratio, skin, lesion, diagnostic, diagnosis, keratinocyte carcinoma

## Introduction

Given the prevalence of keratinocyte carcinomas (KCs), it is imperative to identify accurate diagnostic tools for evaluating suspicious skin lesions [1,2]. Misdiagnosis carries significant harms, including unnecessary scarring, anxiety, and increased cost [3].

## Methods

A 2018 Cochrane review [3] assessed dermoscopy as an adjunct to visual inspection (VI) for KC diagnosis among adults with skin lesions suspicious for malignancy or at risk of KC development [3]. Diagnosis was verified by histology for all malignant lesions, while clinical follow-up or histologic diagnosis was required for at least 50% of participants with benign lesions to be included in the review [3]. When these parameters were met, cancer registry and “expert opinion” were also allowed as reference standards, although this was considered less desirable [3].

## Results

The review [3] included 24 studies conducted between 1987 and 2016, encompassing adult participants from North America, the Middle East, Europe, Oceania, and East Asia. Table 1 presents further information about the included studies. Among the included studies, there were a total of 8805 visually inspected lesions and 6855 lesions inspected with dermoscopy and VI. Face-to-face and teledermatology settings were evaluated separately, although no clear difference was found between settings.

For in-person basal cell carcinoma (BCC) diagnosis, the diagnostic odds ratio revealed dermoscopy and VI were 8.2 (95% CI 3.5-9.3) times more effective than VI alone (likelihood-ratio test *P*<.001), supporting the predicted sensitivity difference of 14% (79% vs 93%) at a fixed specificity of 80% and predicted specificity difference of 22% (77% vs 99%) at a fixed sensitivity of 80%. The predicted values for sensitivity and specificity were estimated using summary receiver operating characteristic (SROC) curves, which were constructed based on data points derived from individual studies included in the review [4]. It is crucial to note that secondary to substantial heterogeneity between studies, the reported differences in sensitivity and specificity are illustrative examples of the values that might be achieved based on the observed data and do not necessarily reflect how the tests might perform in specific settings.

Sources of heterogeneity were unclear due to poor reporting and lack of available data, although the authors suggest that observer experience, type of dermatoscope used, and the case mix of included lesions may have contributed. Risk of bias and concerns regarding applicability were generally high or unclear across most domains assessed, particularly in participant selection, flow, and timing. Although the strength of the conclusions was limited, the addition of dermoscopy to in-person evaluations increased diagnostic accuracy on average. To estimate the impact of the predicted differences in specificity and sensitivity derived from the SROC curve for lesions inspected in person with VI alone versus VI and dermoscopy for the detection of BCC, they were applied to a hypothetical cohort of 1000 lesions. At the median prevalence of 17%, an additional 24 BCC would be identified and 183 fewer non-BCC would be treated unnecessarily with the use of dermoscopy and VI. This information is further illustrated in Table 2. Insufficient data were available for thorough analysis of cutaneous squamous cell carcinoma detection, and it could not be determined whether evaluator expertise or use of a formal algorithm improved the accuracy of KC detection.

**Table 1:** Quantity of evidence for target lesions.

Setting and test (number of studies)			Total lesions, n	Total cases, n
**Basal cell carcinoma quantity of evidence (n=21)**				
	**In person**			
		VI^a^	7017	1586
		VI + D^b^	4683	363
	**Image based**			
		VI	853	156
		VI + D	2271	737
**Cutaneous squamous cell carcinoma quantity of evidence (n=4)**				
	**In person**			
		VI	2684	538
		VI + D	—^c^	—
	**Image based**			
		VI	—	—
		VI + D	717	119
**Any skin cancer quantity of evidence (n=11)**				
	**In person**			
		VI	3618	2021
		VI + D	277	85
	**Image based**			
		VI	517	124
		VI + D	1526	847

^a^VI: visual inspection.

^b^VI + D: visual inspection and dermoscopy.

^c^Not applicable.

**Table 2:** Extrapolation of estimated sensitivity and specificity differences applied to a hypothetical cohort of 1000 lesions^a^.

		Sensitivity^a^			Fixed specificity^b^			Fixed sensitivity^b^			Specificity^c^	
		True positive, n	False negative, n	False positive, n		True negative, n	True positive, n		False negative, n	False positive, n		True negative, n
**10% prevalence**		—^d^	—	180		720	80		20	—		—
	VI^e^	79	21	—		—	—		—	207		693
	VI + D^f^	93	7	—		—	—		—	9		891
**17% prevalence**		—	—	166		664	136		34	—		—
	VI	134	36	—		—	—		—	191		639
	VI + D	158	12	—		—	—		—	8		822
**53% prevalence**		—	—	94		376	424		106	—		—
	VI	419	111	—		—	—		—	108		362
	VI + D	493	37	—		—	—		—	5		465

^a^The dermoscopy test had a sensitivity of 79%, and the visual inspection and dermoscopy test had a sensitivity of 93%.

^b^Both tests had a fixed specificity and fixed sensitivity of 80%.

^c^The dermoscopy test had a specificity of 77%, and the visual inspection and dermoscopy test had a specificity of 99%.

^d^Not applicable.

^e^VI: visual inspection.

^f^VI + D: visual inspection and dermoscopy.

## Discussion

Recent advancements in learning algorithms using dermoscopic images, particularly deep learning techniques like convolutional neural networks (CNNs), have shown promise in improving diagnostic accuracy. In a systematic review [5] of 19 studies conducted between 2017 and 2021, CNNs demonstrated comparable or improved diagnostic accuracy compared to dermatologists. However, it is important to note that these studies primarily focused on melanoma due to its significant risk, leaving a gap in research specifically targeting KCs. Further research dedicated to KC diagnosis is crucial for a comprehensive evaluation of these conditions.

The authors of the review [3] postulated that adjunctive dermoscopy may aid specialists in identifying BCC. However, the results should be considered suggestive rather than conclusive, given the marked heterogeneity and concerns about the methodological quality of the included studies. Further investigation is required to determine any definitive benefit of dermoscopy for BCC diagnosis. Clear identification of evaluator expertise is essential to ensure meaningful results. Moreover, additional evaluation of the use of formal algorithms may benefit clinicians in varying levels of care. The ubiquity of KCs and risks of misdiagnosis underscore the need for transparent reporting of future studies to optimize diagnostic tools and improve outcomes for patients with suspicious skin lesions.

## Abbreviations

BCC: basal cell carcinoma
CNN: convolutional neural network
KC: keratinocyte carcinoma
SROC: summary receiver operating characteristic
VI: visual inspection
